# Weekly laboratory turn-around time identifies poor performance masked by aggregated reporting

**DOI:** 10.4102/ajlm.v9i1.1102

**Published:** 2020-12-21

**Authors:** Lindi-Marie Coetzee, Naseem Cassim, Deborah K. Glencross

**Affiliations:** 1 National Health Laboratory Service (NHLS), Johannesburg, South Africa; 2 Department of Molecular Medicine and Haematology, University of the Witwatersrand, Johannesburg, South Africa

**Keywords:** CD4, turnaround time, laboratory performance, outliers, weekly reporting

## Abstract

**Background:**

High-level monthly, quarterly and annual turn-around time (TAT) reports are used to assess laboratory performance across the National Health Laboratory Service in South Africa. Individual laboratory performances are masked by aggregate TAT reporting across network of testing facilities.

**Objective:**

This study investigated weekly TAT reporting to identify laboratory inefficiencies for intervention.

**Methods:**

CD4 TAT data were extracted for 46 laboratories from the corporate data warehouse for the 2016/2017 financial period. The total TAT median, 75th percentile and percentage of samples meeting organisational TAT cut-off (90% within 40 hours) were calculated. Total TAT was reported at national, provincial and laboratory levels. Provincial TAT performance was classified as markedly or moderately poor, satisfactory and good based on the percentage of samples that met the cut-off. The pre-analytical, testing and result review TAT component times were calculated.

**Results:**

Median annual TAT was 18.8 h, 75th percentile was 25 h and percentage within cut-off was 92% (*n* = 3 332 599). Corresponding 75th percentiles of component TAT were 10 h (pre-analytical), 22 h testing and 1.6 h review. Provincial 75th percentile TAT varied from 17.6 h to 34.1 h, with three good (*n* = 13 laboratories), four satisfactory (*n* = 24 laboratories) and two poor performers (*n* = 9 laboratories) provinces. Weekly TAT analysis showed 12/46 laboratories (28.6%) without outlier weeks, 31/46 (73.8%) with 1–10 outlier weeks and 3/46 (6.5%) with more than 10 (highest of 20/52 weeks) outlier weeks.

**Conclusion:**

Masked TAT under-performances were revealed by weekly TAT analyses, identifying poorly performing laboratories needing immediate intervention; TAT component analyses identified specific areas for improvement.

## Introduction

In South Africa, public health facilities across 52 districts provide patient care through primary healthcare (PHC) services, district, regional and tertiary hospitals. A wide spectrum of tests can be requested and submitted to the nearest pathology laboratory of the National Health Laboratory Service (NHLS). The NHLS is the choice laboratory service provider of the South Africa National Department of Health (NDoH). A network of more than 266 laboratories are strategically placed around the country to optimally accommodate the needs of local communities (urban and rural).^1,2^ Routine laboratory tests have a predetermined total turn-around time (TAT) cut-off that ensures that tests are processed within the required timeframe to effect the appropriate clinical intervention. Total TAT is defined as the time from first registration of a sample on the laboratory information system (LIS) to the time a result is reviewed and released to the requesting physician. TAT is in part determined by (1) the window of testing from venepuncture as prescribed by the test manufacturers, (2) time validity of sample integrity (e.g. how many hours or days before erythrocytes in blood samples die and cannot bind to antibodies effectively) and (3) the clinician timeline (emergency or quick-resulted laboratory testing vs routine laboratory testing).^3,4,5^ Laboratory TAT reflects the time taken for processing a sample and is a direct indicator of laboratory performance and an integral measure of efficiency where delays can impact patient management.^3,4,5,6,7^

HIV-associated tests like HIV viral load (VL) and CD4 counts, like all NHLS laboratory tests, have strict predetermined organisational TAT cut-offs, set to reflect treatment guidelines requirements and standards of care for HIV management by local authorities and the World Health Organization (WHO)^8,9^. These guidelines call for the availability of a CD4 result within 7–14 days.^9^ To meet this standard, the NHLS has set a within-organisation standard of 40 h for 90% of all CD4 testing to be completed and results released.

The accurate reporting of TAT depends on the quality of data collected through the LIS, that is, the inclusion of automated system date and timestamps at various time points in the journey from patient venesection to result review.^10^ For samples sent to NHLS laboratories, four major date and timestamps are electronically collected and used to calculate TAT components^2,10^: (1) pre-analytical time (lab-to-lab), that is, the time from first registration at any NHLS source laboratory to referral receipt at the designated testing laboratory, (2) analytical time (reg-to-test), time from registration at the testing laboratory to result transmission to the LIS, and (3) post-analytical time (test-to-review), time from test transmission onto the LIS to result review and verification by a senior laboratory staff member (i.e. results become available for the requesting physician or nurse to access). Total TAT is the summation of all three TAT components. The limitation of current TAT reporting is that it commences when a sample is registered at a source laboratory (nearest to testing site), that is, the time lapse from patient venesection to sample arrival at the laboratory is not included in the pre-analytical TAT.^10^ The majority of CD4 samples tested in NHLS laboratories originate from public health clinics of the NDoH, with no electronic system linked to LIS. For true clinical TAT assessment and impact on patient care, the time from sample collection to result receipt should be tracked, but this remains a challenge.^11^

Laboratory test TAT in the NHLS is monitored at national, provincial and laboratory level, with annual,^12^ quarterly and monthly reports generated routinely.^10^ Traditionally, the mean TAT is reported, but retrospective data analysis confirmed a non-Gaussian TAT distribution.^4^ Taking this into account, CD4 TAT reporting was upgraded to include the median, 75th percentile and percentage within cut-off to better reflect performance. The concept of classifying laboratory performance was also developed: laboratories are reported as good, satisfactory or poor, based on the 75th percentile and percentage within TAT cut-off value quadrants as described in a recent publication.^10^ The current monthly reports are effective at giving management a snapshot of the CD4 programme and overall (global) laboratory performance^10^ but cannot be used for timely interventions. Underlying problems with TAT are not detected in real time, thus corrective actions are taken retrospectively, days or weeks after they occurred.^10^ More frequent reporting was thus recommended in addition to traditional TAT reporting to enable more meaningful and timely interventions to improve laboratory performance. Using these described TAT parameters and classification, a weekly TAT dashboard was developed and rolled out nationally in 2018 for monitoring the TAT of some tests (the most requested HIV, tuberculosis and non-communicable diseases tests). Turn-around time data will inform corrective action such as additional test operator training.^13,14^ Although the cut-off values for CD4 testing changed from 85% within 48 h to 90% within 40 h in the 2016/2017 financial year, the concept and wording of laboratory performance classification were retained as managers were well acquainted with these terms.

The aim of this article is to describe how weekly review of CD4 TAT analysis can enable the identification of non-compliant laboratories to facilitate effective and timely corrective action and ensure continuous quality management for improved service delivery. Data analysed represents performance prior to the national implementation of the weekly TAT dashboard.

## Methods

### Ethical considerations

Ethics clearance was obtained from the University of the Witwatersrand (M1706108). No patient identifiers were used for this study and laboratories and provinces were anonymised.

### CD4 turn-around time data

CD4 TAT data were extracted from the corporate data warehouse for the financial period April 2016 to March 2017 (2016/2017 financial year) for 46 CD4 testing laboratories. Total TAT was calculated for each sample tested and reported for 52 weeks, together with the TAT component data.

Data analysis included the calculation of the median, 75th percentile and the percentage of samples with a TAT within the stipulated organisation cut-off per week. This was reported per laboratory and per province (aggregated data of laboratories within each of the nine provinces). Performance classification was introduced at provincial level and based on the percentage samples within TAT cut-off as follows: (A) ≥ 95%: good performance, (B) 90.0% – 94.9%: satisfactory performance; (C) 85.0% – 89.9%: moderate to poor performance and (D) 80.0% – 84.99%: poor performance. Performance thus refers to the degree of compliance with NHLS TAT cut-off. The number of weeks that provinces and laboratories did not achieve the 40 h cut-off was reported. Outlier weeks were defined as weeks where the total TAT of all samples tested did not achieve 90% with a TAT under 40 h. Additional data analysis was done on the weekly laboratory data to describe the TAT component contribution to total TAT per laboratory per week and included: (1) lab-to-lab TAT, (2) reg-to-test TAT and (3) test-to-review TAT. The target times set for each TAT component are (1) 14 h, (2) 24 h and (3) 2 h. Although TAT component analysis by laboratory is distributed weekly, for this study, only specific laboratories were selected to represent different levels of compliance and performance categories to demonstrate how individual TAT components affects total TAT. Outlying laboratory TAT components (> 24 h and < 2 h) were correlated with Beckman Coulter engineer logs to verify the impact of instrument downtime on prolonged TAT (data not shown).

Laboratory site visits were conducted to assess root cause analysis for below standard TAT (< 90% processed for > 40 h) performance identified.

### Statistical analysis

Data were prepared and analysed using SAS version 9.4 (Cary, North Carolina, United States) and GraphPad software (San Diego, California, United States). The nine provinces were numbered 1–9, with individual laboratories within a province assigned a number and labelled accordingly (i.e. 1.5 represents province 1 and laboratory 5). Box and whisker plots were created for individual laboratory data over 52 weeks. National total test volumes and TAT was plotted against the 50th and 75th percentiles in a bar graph. Provincial total TAT was plotted as 75th percentile per performance category per week. Individual laboratory distribution of 75th percentiles per performance category was plotted, indicating high (> 350 samples per day), medium (150-350 samples per day) and low volume facilities (< 150 samples per day). Component TAT was plotted as stacked bar graphs, showing the 75th percentile for pre-analytical, testing, and review TAT for selected laboratories representing the four performance categories.

## Results

### Global annual CD4 total turn-around time overview

In this study 3 332 599 CD4 test TAT were analysed. For the 2016/2017 financial year, the national median TAT for all CD4 tests was 18 h with a 75th percentile of 23 h (Table 1). Overall, 91% of all samples met the stipulated cut-off of 40 h, indicating good overall laboratory performance for meeting organisational criteria for CD4 testing. The matched organisational component median (and TAT 75th percentiles) for lab-to-lab TAT was 6.3 (10 h), reg-to-test TAT was 17.3 (22 h) and test-to-review TAT was 1.3 (2.1 h).

**TABLE 1:** National annual National Health Laboratory Service CD4 total turn-around time for all samples tested during the 2016/2017 financial year.

Laboratory TAT component	Total TAT		Target TAT (hours)	75th percentile minimum to maximum
	Median	75th percentile		
Total TAT	18.8	23.0	40	10–49
Laboratory-to-laboratory	6.3	10.0	14	0–24
Registration-to-test	17.3	22.0	24	9–43
Test-to-review	1.3	1.6	2	0–8

TAT, turn-around time; CD4, cluster of differentiation 4.

### National weekly total turn-around time

**FIGURE 1:**
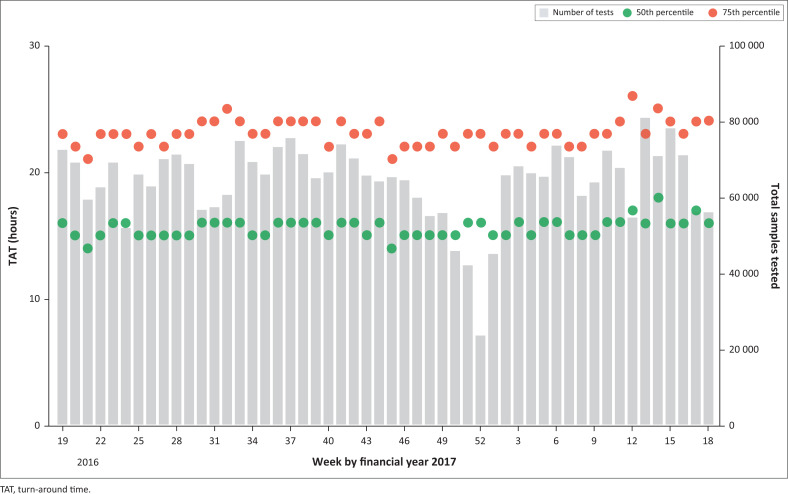
National total turn-around time of National Health Laboratory Service CD4 tests per week for the 2016/2017 financial year. The 50th percentile (median, green circles), 75th percentile (red circles) and volume of samples (grey bars) are depicted.

The weekly distribution of the 50th percentile (median) and 75th percentile showed good consistency despite fluctuations in test volumes across the network of testing laboratories (*n* = 52) (Figure 1). The median ranged from 14 h to 18 h, while the 75th percentile ranged from 21 h to 26 h (Figure 1). Test volumes fluctuated between 23 681 to 80 821 per week (mean of 64 088 tests weekly).

### Provincial total turn-around time distribution (75th percentile) per testing week

Annual global TAT distribution did not identify any poor performance over 52 weeks. To identify poor performances, national TAT were analysed per province. The number of CD4 tests ranged from 65 395 (lowest) to 1 066 137 (highest) (Table 2). The percentage of samples tested within the 40-h TAT cut-off ranged from 82% to 98%. Provincial performance classification was made based on the latter percentage per province, as A to D (as described above). A minimum of three laboratories represented each province.

**TABLE 2:** Annual provincial National Health Laboratory Service CD4 data, indicating test volumes, the 75th percentile total turn-around time, the percentage of samples within turn-around time cut-off and the number of representative laboratories for 2016/2017 financial year.

Province	Number of CD4 tests (52 weeks)	Total TAT		% samples within 40 hour TAT cut-off	Classification based on % samples within TAT cut-off (Group)	Number of CD4 laboratories per province
		75th percentile	Min to max range (hours)			
1	352 277	23.02	18–37	92	90.0–94.9 (B)	6
2	181 391	25.14	16–35	90	90.0–94.9 (B)	3
3	756 661	21.75	20–27	95	≥ 95 (A)	6
4	1 066 137	23.38	21–28	93	90.0–94.9 (B)	11
5	235 772	34.18	18–80	82	80.0–84.9 (D)	4
6	280 985	28.80	20–43	87	85.0–89.9 (C)	5
7	65 395	17.58	13–23	98	≥ 95 (A)	3
8	172 295	18.85	14–40	94	90.0–94.9 (B)	4
9	221 686	20.87	17–27	95	≥ 95 (A)	4

Performance classification: A: good performance; B: satisfactory performance; C: moderate to poor performance; D: markedly poor performance.

TAT, turn-around time; CD4, cluster of differentiation 4; Min, minimum; max, maximum.

Three provinces (3, 7 and 9) were classified as category A (good performance). These laboratories were able to maintain all CD4 reporting within organisation-stipulated TAT at greater than 95% and 75th percentile TAT of 21.7 h (province 3), 17.6 h (province 7) and 2.08 h (province 9). Four provinces (1, 2, 4 and 8) were categorised B (satisfactory performance) having 90% – 94.9% of samples meeting the TAT cut-off; 75th percentile values for these provinces were 23 h, 25.1 h, 23.4 h and 18.9 h, respectively. Two provinces failed to meet the TAT cut-off and were classified as categories C (moderate poor performance; province 6) and D (markedly poor performance province 5), indicating that less than 90% of samples met the CD4 TAT. Within the latter provincial performance clusters (C and D), the 75th percentile reported was 28.8 h and 34.2 h.

**FIGURE 2:**
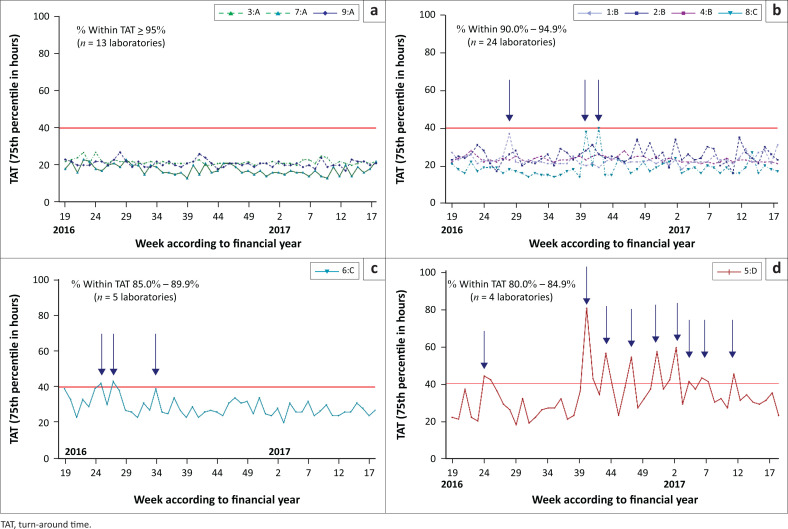
Weekly national 75th percentile CD4 turn-around time of nine provinces per performance category for the 2016/2017 financial year. (a) good performance (*n* = 3 provinces); (b) satisfactory performance (*n* = 4 provinces); (c) moderate to poor performance (*n* = 1 province) and (d) markedly poor performance (*n* = 1 province).

No weekly outliers (weeks where total TAT did not meet 90% < 40 h) were noted in the three good performance provinces (3, 7, and 9; comprising *n* = 13 individual laboratories; Figure 2a). The 75th percentile total TAT for these provinces never exceeded 30 h during the test period. Figure 2b describes the four provinces with satisfactory performance (1, 2, 4 and 8, representing 24 individual laboratories; Table 2). Among this group, province 2 and 4 showed better consistency, easily meeting the TAT cut-off throughout the test period. Province 1 had a week with 75th percentile value of 37 h while Province 8 had two weeks with 75th percentile values of 38 h and 40h. Figure 2c represents Province 6 comprising five laboratories, categorised as a moderate or poor performer, due to inconsistency, especially during weeks 24–34 of 2016, with two weeks having a 75th percentile TAT of over 40 h. After week 35 of 2016, performance stabilised and 75th percentile values corrected to within cut-off values. One province (four individual laboratories; Figure 2d) was classified as a markedly poor performer and characterised by inconsistency and repeated failure to meet the cut-off with less than 85% of reported tests meeting stipulated organisational TAT cut-off (10 weeks exceeding 40 h).

### Individual laboratory total turn-around time by week and performance category

**FIGURE 3:**
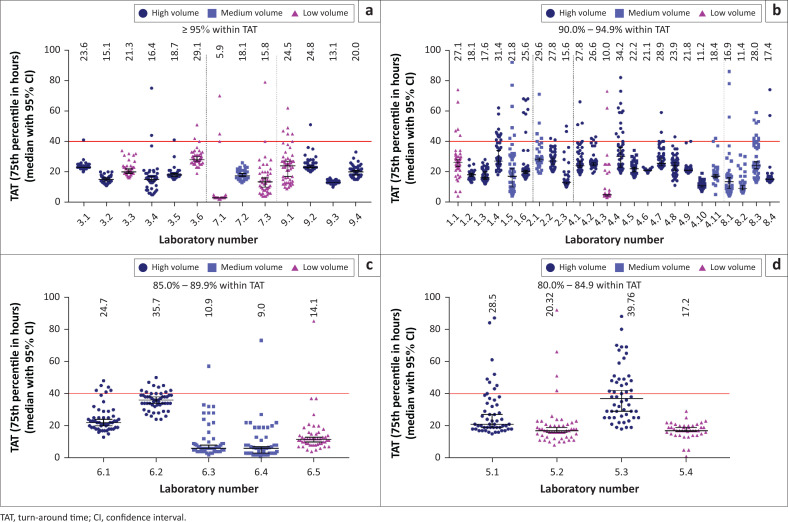
Scatter plots of the 75th percentile total turnaround time of individual laboratories in the National Health Laboratory Service within the provincial performance classification groups A to D for the 2016/2017 financial year. The overall 75th percentile total turn-around time per laboratory is indicated above each plot. (a) good performance (*n* = 13 laboratories); (b) satisfactory performance (*n* = 24 laboratories), (c) moderate to poor performance (*n* = 5 laboratories), and (d) markedly poor performance (*n* = 4 laboratories). High-volume (blue circles), medium volume (light blue squares) and low-volume (pink triangles) laboratories are indicated. The red line in each graphs represents the target total TAT. Individual median total TAT is indicated above each representative laboratory.

The different performance levels identified at provincial level still masked the performance and contribution of individual laboratories to provincial performance. Scatter plots were constructed to visualise the performance of individual laboratories over 52 weeks per provincial performance category. Results showed that irrespective of the provincial performance classification (Figures 3a–d), individual laboratory performance included good, satisfactory and poor performance laboratories.

The 75th percentile across 52 weeks for the good performance provinces (3 provinces and 13 laboratories) showed good overall compliance (tight clumping of weekly 75th percentile values) where the overall 75th percentile for the whole period ranged from 5.9 h (laboratory 7.1) to 24.8 h (laboratory 9.2) (Figure 3a). Similarly, the satisfactory performance provinces (*n* = 24 laboratories) (Figure 3b) had a 75th percentile ranging from 10 h (laboratory 4.3) to 34.2 h (laboratory 4.4). The provinces having moderately poor performance had variable TAT between 9 h (laboratory 6.4) to 35.7 h (laboratory 6.2) (Figure 3c) while markedly poor performance provinces had TAT 75th percentile values ranging from 17.2 h (laboratory 5.4) to 39.7 h (laboratory 5.5) (Figure 3d). Overall, four laboratories recorded a 52-week median TAT of more than 30 h.

The number of weeks that laboratories did not meet the cut-off criteria of 90% with TAT under 40 h varied among categories and laboratories (0–22 weeks), where 12 of 46 laboratories (irrespective of performance category) had zero outlying weeks (26%), 25/46 (54%) more than 5 outlying weeks and 6 (13%) between 6 and 10 outlying weeks. Only three laboratories showed outliers for more than 10 weeks where cut-off was not met (6.5%).

### Case examples of individual laboratory component turn-around time analysis

**FIGURE 4:**
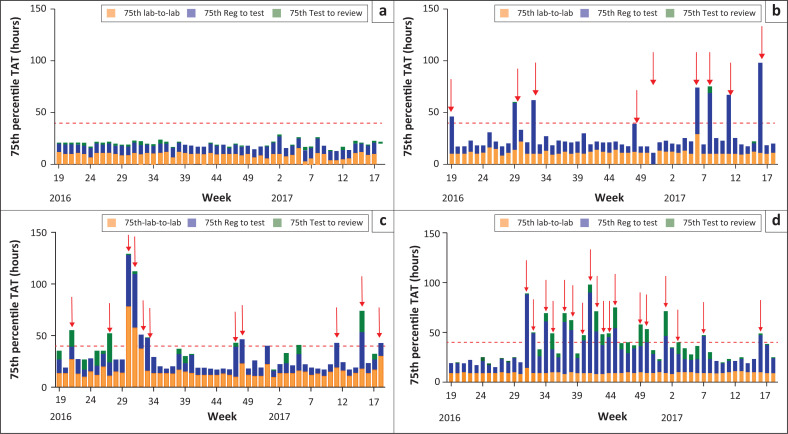
Stacked bar graphs showing examples of individual laboratory weekly performance for 2016/2017 financial year. 75th percentile turn-around time components color-coded: lab-to-lab (orange), reg-to-test (blue) and test-to-review (green), with cut-off of 40 h (red dotted lines). (a) good performance (laboratory A); (b) satisfactory performance (laboratory B); (c) moderate to poor performance (laboratory C) and (d) markedly poor performance (laboratory D).

Figure 4a shows a good performer high-volume laboratory, doing more than 350 samples per day with no outlying weeks (exceeding 40 h cut-off). Over the 52 weeks, 86 559 tests were performed by this laboratory. A lab-to-lab 75th percentile of 9.5 h was reported (ranging from 3 h to 16 h), with a reg-to-test of 8.8 h (range from 6 h to 17 h) and a test-to-review of 1.3 h (range from 0 h to 4 h). More than 98% of all samples tested had a total TAT of under 40 h.

Figure 4b represents a satisfactory performance laboratory, doing 63 998 samples for the period. It reported nine non-consecutive weeks of outliers (exceeding 40 h). Of these outlier weeks, eight were due to prolonged reg-to-test (testing delay), where this component contributed between 27 h and 87 h to the total TAT reported for these weeks. One week (week 51 of 2016) had an extended lab-to-lab value of 65 h, due to confirmed challenges with logistics. Test-to-review ranged from 0 h to 6 h and, as such, had no contribution to the outlying weeks. Extended reg-to-test (within laboratory TAT) seen in weeks 6–11 of 2017 correlated with instrument downtime based on data provided by Beckman Coulter call centre log on engineers dispatched.

The laboratory represented in Figure 4c (moderate to poor performance) tested 77 640 samples during the 52 weeks and had an overall lab-to-lab 75th percentile of 17.7 h (ranging from 10 h to 78 h per week), with 11 weeks exceeding the target total TAT (highest recorded total TAT of 129 h). The lab-to-lab component (orange bars) contributed to total TAT outlying weeks during weeks 21, 30–32, and 48 of 2016 and weeks 11 and 18 of 2017. Reg-to-test (within laboratory TAT) was the leading cause of outliers noticed during weeks 30–32, 47–48 of 2016 and weeks 11, 15 and 18 of 2016. The combined extended TAT in two components (i.e. pre-analytical or lab-to-lab and analytical or reg-to-test) for weeks 30–32 of 2016 and weeks 15 and 18 of 2017 contributed to the total TAT for this laboratory to fall into a category of 85% – 90% of samples within the TAT target of 40 h.

The laboratory contributing the most outlying weeks to group D (Figure 3d) was analysed for component TAT. This laboratory had 18 weeks of exceeding the target total TAT. This was for the most part due to prolonged within laboratory TAT (blue bars, Figure 4d). From week 31 to 45 of 2016 the reg-to-test component contributed as much as 81 h (week 40 of 2016) to the weekly TAT. During this period, some weeks also experienced prolonged test-to-review times of up to 25 h (week 1 of 2017).

## Discussion

TAT remains a key performance indicator of laboratory service efficiency.^4,14^ The parameters reported (mean vs median) and time intervals of reporting impacts the utilisation of TAT as a means to identify and address non-compliance to organisational cut-offs. Definitions of TAT may vary and depend on the test (routine or emergency), priority of reporting (immediate or delayed clinical intervention), the population served and activities or components measured.^4^ The clinical outcome, needs and responsibilities of management determine how TAT information is used to ensure that there are no unnecessary delays in result reporting. Across the NHLS, TAT information typically remains the jurisdiction of the testing laboratory where the laboratory manager uses this data to identify problems and initiate corrective action; the individual laboratory has sole and direct access to its own daily or weekly TAT data.^3,4,5^ TAT monitoring is however critical for priority programmes, such as HIV and tuberculosis,^2,12^ where individual laboratories monitor their respective test TAT, while the organisation is responsible for reporting performance of the network of laboratories. Relevant updated information on the efficiency of service delivery is vital in this context for risk assessment and timely intervention to ensure the continued excellence of service delivery^10^ and meeting dire local HIV and tuberculosis programme needs.^9^

Ideally, sample-by-sample real-time reporting of TAT would be the preferred way to monitor and assist laboratories in the identification of specific service delivery and related TAT challenges. Hierarchical global overview (usually annual) TAT reporting is however the simplest and most widely used, but masks poor performance, as confirmed by data from this study. Interrogating the weekly data by drilling down to laboratory level at weekly intervals, enables the identification of outliers and poor performers. This study showed that lower hierarchical levels, as well as shorter time periods, can unveil problematic and inefficient testing laboratories (Figure 2 and Figure 3). Adding weekly TAT component analysis at laboratory level further identifies problematic testing weeks and possible causes of prolonged TAT (Figure 4 and Box 1).

BOX 1 Summary of study findings.

- National total TAT and TAT component results paint a picture of good overall performance for a specific test (i.e. CD4), masking underlying performance outliers.
- Analysing smaller sections of data (province and laboratory) for shorter test periods (month and week) reveals the true performance level based on compliance with organisational test cut-offs.
- Performance can be classified as good, satisfactory or poor based on the percentage of samples within the test cut-off and the mean 75th percentile total TAT reported.
- Weekly TAT component analysis at laboratory level reveals possible causes of delayed total TAT and provides some insight into the type of intervention required.

TAT, turn-around time; CD4, cluster of differentiation 4.

The most common issues that impacted on prolonged lab-to-lab (pre-analytical) TAT were delays in transport or sample collection from clinics to testing laboratories and changes in courier routes and pick up times. Reg-to-test times (laboratory TAT) were prolonged due to instrument downtime, lack of trained staff to operate testing instruments, delayed sample registration in the testing facility, challenges with reagent availability and timely delivery, and staff strikes.^15^ Delays in the test-to-review phase, were mostly due to lack of result auto-authorisation in high-volume laboratories, nonavailability of result authorising staff when samples are analysed during night shifts or where problems with connectivity of the TrackCare LIS were reported. Several references focus on the main causes of TAT delays.^5,16,17,18,19,20,21,22^ The main objective of this study, however, was not to describe causes of delayed TAT, but to emphasise the importance of TAT monitoring with shorter time intervals as a means of more proactive interventions for sustained good performance across network of laboratories.

Data reported here demonstrate the need for more frequent TAT reporting in effective performance management.^10,13,15,23,24^ An interactive weekly TAT dashboard was introduced nationally in 2018 for the most requested tests across the NHLS. Frequent performance reporting can be effective in identifying challenges with meeting target TAT cut-offs allowing for timely interventions.^13^ Weekly assessment of TAT and TAT components not only identifies problematic testing laboratories or days with TAT challenges, but also enables the identification of individual outlier samples that can be investigated (root cause analysis) to assess causes of TAT delays.

Based on the data presented in this study, further refinement of the current reporting platforms is recommended to include daily reporting for rapid proactive intervention. A further recommendation is to integrate daily quality control tests, external quality assessment testing and equipment downtime supplier call-out data into the reporting to assist with focused troubleshooting and interventions.

Turn-around time monitoring and reporting are however guided by the requirements of the end user and will continue to be available at various time intervals for laboratory network management to assess overall trends, with weekly or daily reports to laboratory and programme managers enabling timely proactive intervention to ensure optimal laboratory performance and timely patient result reporting.

### Limitations

The monitoring of TAT in the NHLS is currently limited as end-to-end sample tracking system is absent. The TAT reported in this article thus only represents the time from first registration on the LIS to review of the result.

### Conclusion

National and provincial analysis of TAT mask individual laboratory performance; therefore, TAT analysis by week and by laboratory is recommended to highlight laboratory TAT inefficiencies. Root-cause analyses were able to identify pre-analytical, analytical or post-analytical factors contributing to performance. TAT data was used to categorise performance at the provincial and laboratory level.

This study used the concept of zooming in to lower levels and shorter times of TAT reporting to identify possible non-compliant laboratories. In conclusion: (1) outlying weeks are not prescribed by provincial or laboratory classification of performance, (2) performance did not correlate to the size of the laboratory (i.e. test volumes of high, medium and low), (3) there were laboratories that had no outlier weeks during the analysed period that can serve as model laboratories for setting performance standards and good reproducibility of week-on-week performance across a network of testing laboratories.

## References

[1] National Health Laboratory Service (NHLS) NHLS annual report 2014/15 Sandringham [homepage on the Internet]. Johannesburg: National Health Laboratory Service; 2015 [cited 2017 Jul 26]. Available from: http://intranet.nhls.ac.za/assets/files/policy/NHLS_Annual_Report_2015.pdf

[2] Glencross DK, Coetzee L, Cassim N An integrated tiered service delivery model (ITSDM) based on local CD4 testing demands can improve turn-around times and save costs whilst ensuring accessible and scalable CD4 services across a national programme. PLoS One. 2014;9(12):e114727 10.1371/journal.pone.0114727 25490718 PMC4260875

[3] Goswami B, Singh B, Chawla R, Gupta VK, Mallika V Turn around time (TAT) as a benchmark of laboratory performance. Indian J Clin Biochem. 2010;25(4):376–379. 10.1007/s12291-010-0056-4 21966108 PMC2994570

[4] Hawkins RC Laboratory turnaround time. Clin Biochem Rev. 2007;28(4):179–194. 18392122 PMC2282400

[5] Pati HP, Singh G Turnaround time (TAT): Difference in concept for laboratory and clinician. Indian J Hematol Blood Transfus. 2014;30(2):81–84. 10.1007/s12288-012-0214-3 24839360 PMC4022919

[6] Valenstein P Laboratory turnaround time. Am J Clin Pathol. 1996;105(6):676–688. 10.1093/ajcp/105.6.676 8659441

[7] Valenstein PN, Emancipator K Sensitivity, specificity, and reproducibility of four measures of laboratory turnaround time. Am J Clin Pathol. 1989;91(4):452–457. 10.1093/ajcp/91.4.452 2929500

[8] World Health Organisation Guidelines for managing advanced HIV disease and rapid initiation of antiretroviral therapy [homepage on the Internet] Policy Brief. Geneva: WHO; 2017 [cited 2019 Jul 16]. Available from: https://www.who.int/hiv/pub/toolkits/advanced-HIV-disease-policy/en/29341560

[9] National Department of Health (NDOH) National consolidated guidelines for the prevention of mother-to-child transmission of HIV (PMTCT) and the management of HIV in children, adolescents and adults [homepage on the Internet]. Pretoria; 2015 [cited 2019 Jul 16]. Available from: https://sahivsoc.org/Files/ART%20Guidelines%2015052015.pdf

[10] Coetzee L, Cassim N, Glencross DK Using laboratory data to categorise CD4 laboratory turn-around-time performance across a national programme. Afr J Lab Med. 2018;7(1):a665 10.4102/ajlm.v7i1.665 PMC6111574 30167387

[11] Stotler BA, Kratz A Determination of turnaround time in the clinical laboratory: “Accessioning-to-result” time does not always accurately reflect laboratory performance. Am J Clin Pathol. 2012;138(5):724–729. 10.1309/AJCPYHBT9OQRM8DX 23086774

[12] National Health Laboratory Service (NHLS) Annual report 2017/18 [homepage on the Internet]. Johannesburg: National Health Laboratory Service (NHLS); 2018 [cited 2018 Dec 10]. Available from: http://www.nhls.ac.za/assets/files/an_report/NHLS_AR_2018.pdf

[13] Cassim N, Coetzee LM, Tepper MEE, Perelson L, Glencross DK Timely delivery of laboratory efficiency information, Part II: Assessing the impact of a turn-around time dashboard at a high-volume laboratory. Afr J Lab Med. 2020;9(2):a948 10.4102/ajlm.v9i2.948 PMC7203269 32391245

[14] Cassim N, Coetzee L-M, Tepper M, Motlonye B, Glencross DK, editors TAT as a risk model for operational services. Johannesburg: PathRed 2017.

[15] Coetzee L, Cassim N, Tepper M, Glencross DK The importance of reporting individual weekly laboratory turn-around-time (TAT) to identify outliers and underperformance masked during global annual TAT review. ASLM Conference; 2018 Dec 10–13; Abuja; p. Poster: ID PS-2.3b-070.

[16] Cakirca G The evaluation of error types and turnaround time of preanalytical phase in biochemistry and hematology laboratories. Iran J Pathol. 2018;13(2):173–178. 10.30699/ijp.13.2.173 30697287 PMC6339495

[17] Chauhan KP, Trivedi AP, Patel D, Gami B, Haridas N Monitoring and root cause analysis of clinical biochemistry turn around time at an academic hospital. Indian J Clin Biochem. 2014;29(4):505–509. 10.1007/s12291-013-0397-x 25298634 PMC4175690

[18] Jalili M, Shalileh K, Mojtahed A, Mojtahed M, Moradi-Lakeh M Identifying causes of laboratory turnaround time delay in the emergency department. Arch Iran Med. 2012;15(12):759–763. https://doi.org/0121512/AIM.008 23199248

[19] Khalifa M, Khalid P Improving laboratory results turnaround time by reducing pre analytical phase. Stud Health Technol Inform. 2014;202:71–74. 10.3233/978-1-61499-423-7-71 25000018

[20] Lou AH, Elnenaei MO, Sadek I, Thompson S, Crocker BD, Nassar BA Multiple pre- and post-analytical lean approaches to the improvement of the laboratory turnaround time in a large core laboratory. Clin Biochem. 2017;50(15):864–869. 10.1016/j.clinbiochem.2017.04.019 28457964

[21] Minchella PA, Chipungu G, Kim AA, et al Specimen origin, type and testing laboratory are linked to longer turnaround times for HIV viral load testing in Malawi. PLoS One. 2017;12(2):e0173009 10.1371/journal.pone.0173009 28235013 PMC5325555

[22] Saathoff AM, MacDonald R, Krenzischek E Effectiveness of specimen collection technology in the reduction of collection turnaround time and mislabeled specimens in emergency, medical-surgical, critical care, and maternal child health departments. Comput Inform Nurs. 2018;36(3):133–139. 10.1097/CIN.0000000000000402 29120913

[23] Cassim N, Tepper ME, Coetzee LM, Glencross DK Timely delivery of laboratory efficiency information, Part I: Developing an interactive turnaround time dashboard at a high-volume laboratory. Afr J Lab Med. 2020;9(2):a947 10.4102/ajlm.v9i2.947 PMC7203318 32391244

[24] Coetzee L, Cassim N, Tepper M, Glencross DK Standardizing individual laboratory turnaround time (TAT) performance amongst laboratories in a CD4 testing network. PathCape 2018: 56th International FSASP Congress; 2018 Aug 16–18; Stellenbosch.

